# An Inflammatory Response-Related Gene Signature Reveals Distinct Survival Outcome and Tumor Microenvironment Characterization in Pancreatic Cancer

**DOI:** 10.3389/fmolb.2022.876607

**Published:** 2022-06-08

**Authors:** Fengxiao Xie, Xin Huang, Chaobin He, Ruiqi Wang, Shengping Li

**Affiliations:** ^1^ Department of Pancreatobiliary Surgery, Sun Yat-sen University Cancer Center, Guangzhou, China; ^2^ Sun Yat-sen University Cancer Center, State Key Laboratory of Oncology in South China, Collaborative Innovation Center for Cancer Medicine, Guangzhou, China

**Keywords:** inflammatory response, immune characteristics, tumor microenvironment, pancreatic cancer, chemotherapy response

## Abstract

**Background:** Desmoplasia or rich fibrotic stroma is a typical property of pancreatic cancer (PC), with a significant impact on tumor progression, metastasis, and chemotherapy response. Unusual inflammatory responses are considered to induce fibrosis of tissue, but the expression and clinical significance of inflammatory response-related genes in PC have not been clearly elucidated.

**Methods:** Prognosis-related differentially expressed genes (DEGs) between tumor and normal tissues were identified by comparing the transcriptome data of PC samples based on The Cancer Genome Atlas (TCGA) portal and the Genotype Tissue Expression (GTEx) databases. Samples from the ArrayExpress database were used as an external validation cohort.

**Results:** A total of 27 inflammatory response-related DEGs in PC were identified. Least absolute shrinkage and selection operator (LASSO) analysis revealed three core genes that served as an inflammatory response gene signature (IRGS), and a risk score was calculated. The diagnostic accuracy of the IRGS was validated in the training (*n* = 176) and validation (*n* = 288) cohorts, which reliably predicted the overall survival (OS) and disease-free survival (DFS) of patients with PC. Furthermore, multivariate analysis identified the risk score as an independent risk factor for OS and DFS. The comprehensive results suggested that a high IRGS score was correlated with decreased CD8^+^ T-cell infiltration, increased M2 macrophage infiltration, increased occurrence of stroma-activated molecular subtype and hypoxia, enriched myofibroblast-related signaling pathways, and greater benefit from gemcitabine.

**Conclusion:** The IRGS was able to promisingly distinguish the prognosis, the tumor microenvironment characteristics, and the benefit from chemotherapy for PC.

## Introduction

Death from pancreatic cancer (PC) is ranked fourth among cancer-related deaths in Western countries, with a low 5-year overall survival (OS) rate (below 10%) (Madurantakam Royam et al., 2019). Presently, surgical resection remains the only chance for cure (Springfeld et al., 2019; Sunami and Kleeff, 2019), but because patients typically present with locally advanced or metastatic disease, less than 20% of newly diagnosed patients are eligible for radical pancreatoduodenectomy due to incurable-stage diagnoses (Mizrahi et al., 2020). Among patients at the unresectable stage, chemotherapy is important for improving survival (Springfeld et al., 2019). Poor prognosis in PC correlates with non-specific symptoms in the early stage, an immunosuppressive tumor microenvironment (TME), and chemotherapy resistance or inefficient screening measures for early detection.

Although recent improvements have been made in chemotherapy and molecular targeted therapies, extensive toxicity and limited survival benefit remain in unselected populations (Cheng et al., 2019). A large body of evidence demonstrates that personalized treatment of this aggressive disease is associated with increased survival and an improved quality of life (Cheng et al., 2019; Wu et al., 2019). For this reason, there is a desperate need for validated models to assess prognosis, with the goal of providing individualized treatments for patients. Furthermore, comprehensive analysis as opposed to targeting a single factor is necessary to discover reliable prognostic biomarkers that can assist in guiding treatment strategies for PC patients.

Accurate staging of PC is essential for prognostic assessment to guide treatment, but frustratingly, the current tumor, node, and metastasis (TNM) staging system is insufficient due to extensive intra-staging heterogeneity (Raman et al., 2018; van Roessel et al., 2018). Recent studies have indicated the diversity and complexity of genetic variation in human PC, which can account for different disease behaviors in clinical settings. To date, a large number of studies have focused on biomarkers in blood and PC tissue for survival prediction. However, most studies have only focused on disparate genes, and this is not sufficiently valid for patient diagnosis or prognosis. Additionally, few screening biomarkers have been validated for clinical application (Harsha et al., 2009; Yachida et al., 2010). Therefore, there may be increased efficacy in the use of a combination of different biomarkers as prognostic indices.

Dense fibrotic stroma comprising most of the neoplastic mass is a remarkable feature of PC, and it is responsible for tumor progression, metastasis, and chemotherapy resistance (Mortezaee, 2021). Therefore, the exploration of targeting the pro-oncogenic stroma in combination with conventional treatments has currently been an emerging strategy. It is believed that a desmoplastic reaction results from an unsupervised injury repair process and abnormal inflammatory responses (Wynn, 2007; Erkan et al., 2012; Kota et al., 2017). Researchers have been interested in the link between inflammatory responses and cancers of different origins for nearly 100 years. It has been shown that an increased inflammation-related prognostic score, the modified Glasgow prognostic score (mGPS) that grades serum albumin and C-reactive protein, is correlated with poor prognosis in cancers (McMillan, 2013). In addition, high systemic inflammatory response indexes, such as the neutrophil–lymphocyte ratio, platelet–lymphocyte ratio, and monocyte–lymphocyte ratio, have been categorically demonstrated to contribute to poor prognosis and chemotherapeutic sensitivity in cancers, including PC (Steele et al., 2016). In addition to the systemic inflammatory response, a local inflammatory response in the TME can promote tumor growth and metastasis by promoting non-normalized angiogenesis and tissue remodeling, presenting an immunosuppressive phenotype in PC (Vonderheide and Bayne, 2013). A recent study has also shown that the combination of local inflammatory cells and systemic inflammatory response analysis can greatly assist in the prognostic estimation and tailor treatment plans for patients with colon cancer (Turner et al., 2016). Nevertheless, comprehensive analysis of inflammatory response-related genes connected to prognosis and TME characteristics of PC has been rarely reported and remains to be elucidated.

To fully understand the function of inflammatory response-related genes in PC, the present study identified differentially expressed genes (DEGs) using a deep bioinformatics analysis based on publicly available databases. Subsequently, OS-related DEGs were identified via Cox regression analyses, and an inflammatory response gene signature (IRGS) was constructed by the least absolute shrinkage and selection operator (LASSO)-Cox method. Enrolled samples from an independent cohort originating from ArrayExpress microarray datasets validated the signature. Independent prognostic factors concerning OS and disease-free survival (DFS) were investigated by multivariate Cox survival analysis. Then, DEGs between different risk groups were tested by pathway enrichment analysis. We also performed a comprehensive exploration of the clinical role, gemcitabine sensitivity prediction, and TME characterization of the IRGS. Overall, this novel inflammatory response gene signature for the prediction of prognosis in PC might serve as a meaningful complement to the traditional TNM staging system for improving prognostic stratification and tailoring individual therapy.

## Materials and Methods

### Acquisition of Raw Data

Transcriptome sequencing data for 178 PC samples and 4 normal pancreas samples and corresponding matched clinical information were downloaded from TCGA PanCancer Atlas. One sample with tumor recurrence and one sample with 0 follow-up days were excluded, and thus, we finally obtained 176 samples in TCGA cohort. The gene expression information for 328 normal pancreas samples was extracted from the Genotype Tissue Expression (GTEx) project for further analysis.

Regarded as an external validation group, the E-MTAB-6134 cohort containing clinical and microarray gene expression data for 309 PC patients was retrieved from ArrayExpress, which is the most commonly used genomic data repository, of which there was complete survival status information for 288 cases. The lists of inflammatory response-related genes were obtained from the Molecular Signatures (https://www.gsea-msigdb.org/gsea/msigdb/cards/HALLMARK_INFLAMMATORY_RESPONSE.html) database.

### Differential Gene Expression Analysis

To accurately obtain DEGs, the DEGs in PC were determined from TCGA and GTEx datasets. RNA sequencing data were calculated as transcripts per million (TPM) values and were log2 (TPM + 1) transformed. Then, the R package “limma” was applied, and the adjusted *p* value (*p*.adj) was calculated in TCGA and GTEx to reduce false-positive discoveries. Genes with *p*.adj < 0.05 and log2 |fold change (FC)| > 2 calculated through gene expression values were considered DEGs.

### Pathway Enrichment Analyses

We performed Kyoto Encyclopedia of Genes and Genomes (KEGG) pathway analysis and gene set enrichment analysis (GSEA) for pathway enrichment analyses. GSEA was carried out between high- and low-risk PC samples to more accurately describe overall changes (Subramanian et al., 2005). Additionally, Gene Ontology (GO) terms and KEGG pathways concentrated in candidate gene lists were identified based on the R package “ClusterProfiler” to conduct the exploration of biological processes, cellular components, and molecular functions (Yu et al., 2012). *p*.adj < 0.05 was considered to be meaningful enrichment.

### Identification of Core Genes and Construction of the Prognosis-Associated Inflammatory Response Gene Signature

Prognosis-related DEGs identified by differential analysis and Cox regression univariate analysis were integrated for LASSO analysis, an extensively used machine learning technique that can optimize the selection of genes and avoid overfitting (Jiang et al., 2018), to construct the IRGS in TCGA cohort. In this analysis, the optimal LASSO tuning parameter (Lambda) was selected as lambda.1se (namely, the value of lambda would result in the lowest partial likelihood deviance value with less than one standard error), and 10-fold cross-validation was performed to obtain a more stable coefficient. The IRGS-related risk score was calculated as follows:

$$\text{Risk}\;\text{score}=\sum_{k=1}^{n}\left(E\text{x}p_{k}\;*\;\beta_{k}\right),$$

where Exp_k_ denotes the expression level of the *k*th gene and β denotes a regression coefficient. Prognosis-related DEGs filtered by LASSO-Cox regression were considered core genes.

To verify the reproducibility of our model, we used the same cutoff value in the subsequent validation analysis. The prognostic power of IRGS for survival was assessed in TCGA and E-MTAB cohorts utilizing receiver-operating characteristic (ROC) curves.

### Evaluation of the TME

We used the CIBERSORT deconvolution algorithm to assess the characteristics of tumor-infiltrating immune cells (TIICs) in tissues according to the gene expression data (Newman et al., 2015). The eligible data (*p* < 0.05) were considered accurate for further analysis. The ESTIMATE score was used to estimate the TME according to a previous study (Chen et al., 2018). We investigated the relationship between the TME and the IRGS.

### Prediction of the Sensitivity of Chemotherapy Drugs

We assessed the response of samples to gemcitabine, a widely used chemotherapy agent in PC, utilizing the “pRRophetic” R package in which the half-maximal inhibitory concentration (IC50) of a sample was predicted by ridge regression according to a previous investigation (Geeleher et al., 2014). All default parameters were used, and duplicate gene expression was summarized as a mean value.

### Data Analysis

The Kruskal–Wallis, Wilcoxon rank-sum, or chi-square tests were applied to determine the statistical relevance between groups. The Kaplan–Meier method and log-rank test were conducted to analyze the correlation between variables and OS and DFS. Cox regression univariate and multivariate analyses were also performed to analyze independent prognostic factors with hazard ratios (HRs). Statistical analysis was performed in R software v3.6.3. *p* less than 0.05 was considered statistically significant.

## Results

### Profile of Prognostic Inflammation-Related Gene Expression in Normal and PC Samples

**FIGURE 1:**
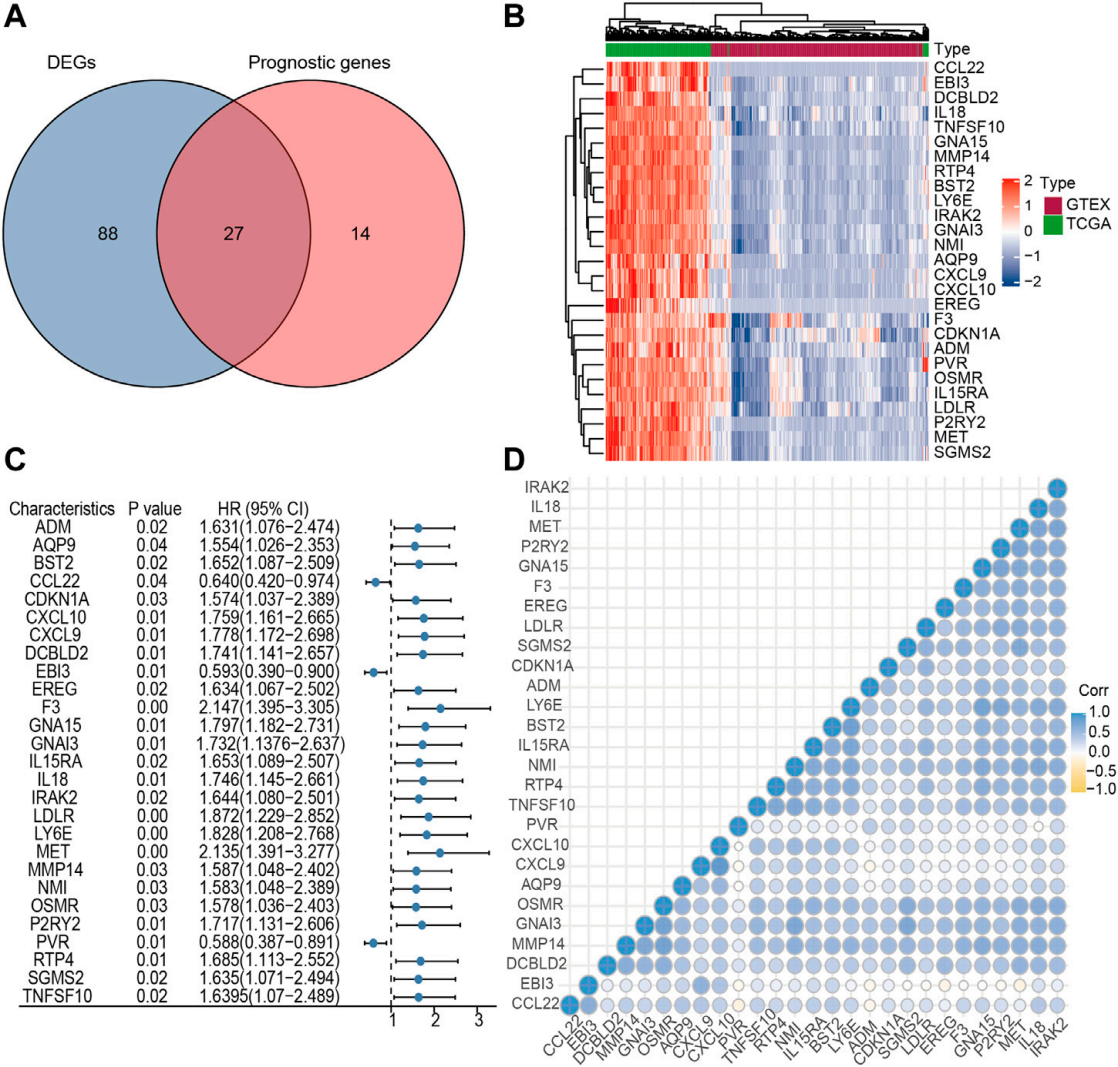
Identification of candidate inflammatory response-related genes in PC. **(A)** Venn diagram of DEGs between TCGA and GTEx. **(B)** Heatmap of prognosis-related DEGs. **(C)** Forest plots of the association between 27 prognosis-related DEGs and OS. **(D)** Cross correlogram between the 27 candidate genes.

The limma algorithm identified 115 DEGs related to inflammatory response in PC between TCGA and GTEx, of which 27 were relevant to OS by single-factor Cox analysis (Figures 1A,B). From this collection of genes, the F3 gene possessed a maximum hazard ratio of 2.147 (95% CI = 1.395–3.305, *p* = 0.00, Figure 1C); the correlation of the 27 genes is shown in Figure 1D. The above results demonstrated a different profile of inflammation-related gene expression between PC and normal samples.

### Screening of Core Inflammation-Related Genes and Construction of Prognostic Signature in TCGA

**FIGURE 2:**
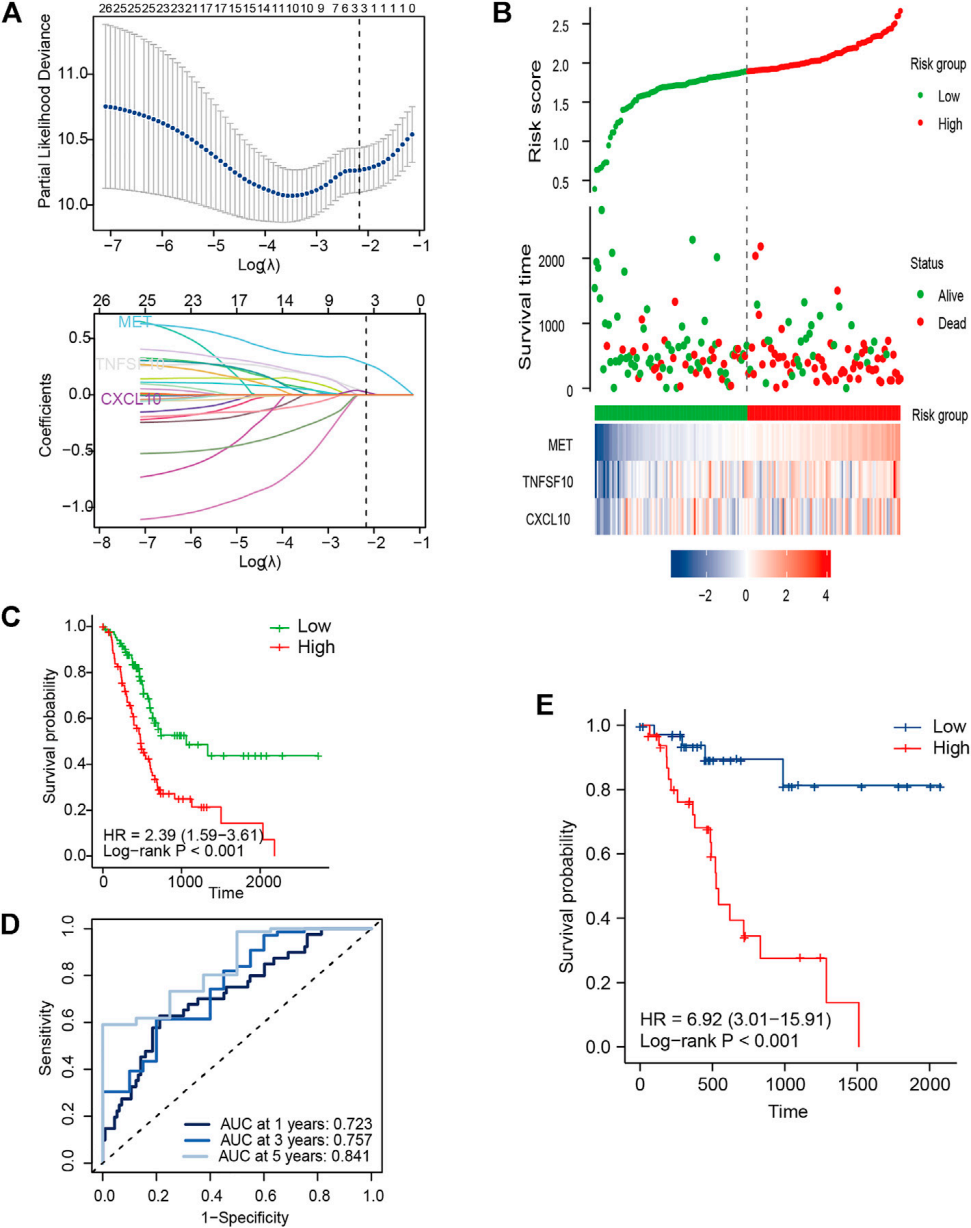
Development of signature genes in TCGA cohort. **(A)** LASSO regression model for selecting the number of parameters and developing the prognosis prediction signature. **(B)** IRGS distribution, live status, and expression heatmap for the three core genes. **(C)** Kaplan–Meier (KM) survival curves for OS for PC patients. **(D)** ROC curve analysis of the IRGS for OS. **(E)** KM survival curves for DFS for PC patients.

Due to an extremely low 5-year survival rate in PC, we selected OS as the clinical outcome. The IRGS consisting of three core genes for prognostication was constructed in TCGA cohort using LASSO-Cox regression analysis (Figure 2A), and for each patient, an IRGS-related risk score was measured by the following formula: Risk score = [(0.2755) × Expression level of MET] + [0.0287 × Expression level of TNFSF10] + [(0.0235) × Expression level of CXCL10]. Based on the total points for the risk score, 176 PC patients without repeat clinical information from TCGA were stratified into two groups using the median risk score value as a cutoff value (cutoff value = 1.89328062). The corresponding risk score, live status, and distribution of TCGA cohort are presented in Figure 2B.

For measuring the prognostic accuracy of the novel IRGS, the area under the ROC curve (AUC) was generated, as well as the Kaplan–Meier survival curve. The results showed that the OS of patients in the high-risk group was significantly shorter than that in the low-risk group (*p* < 0.001; Figure 2C). Additionally, the 1-, 3-, and 5-year OS AUC of IRGS was 0.723, 0.757, 0.841, respectively (Figure 2D), and patients in the high-risk group exhibited a remarkably shorter DFS (Figure 2E). Subsequently, to confirm whether the IRGS was an independent risk feature connected with patients’ OS and DFS, univariate and multivariate Cox regression analyses were performed. The regression results suggested that the tumor site and chemotherapy, as well as the IRGS, were robust independent predictors for OS (IRGS: *p* < 0.001; tumor site: *p* = 0.038; chemotherapy: *p* < 0.001; Table 1). The IRGS remained an independent prediction factor for DFS after adjusting for clinicopathological variables (age, T-staging, tumor site, lymph node metastasis, and chemotherapy) (*p* < 0.001; Table 2). Similarly, our results confirmed the independent predictive role of IRGS for OS and DFS in the E-MTAB cohort (Supplementary Tables 1, 2).

**TABLE 1:** Independent prognostic factors for OS in TCGA dataset.

Characteristic	Total (N)	Univariate analysis		Multivariate analysis	
		Hazard ratio (95% CI)	*p* value	Hazard ratio (95% CI)	*p* value
Risk score	176	7.076 (3.493–14.334)	**<0.001** ^a^	4.874 (2.439–9.742)	**<0.001** ^a^
Age (years)	176				
<65	81	Reference			
≥65	95	1.415 (0.931–2.150)	0.104		
Sex	176				
Male	96	Reference			
Female	80	1.219 (0.809–1.837)	0.343		
N	171				
N0	49	Reference			
N+	122	2.106 (1.254–3.539)	**0.005** ^a^	1.732 (0.164–18.240)	0.648
T	174				
T1+T2	31	Reference			
T3+T4	143	2.040 (1.082–3.850)	**0.028** ^a^	1.443 (0.534–3.902)	0.470
AJCC stage	173				
Stage I	21	Reference			
Stage IIA	28	1.429 (0.551–3.707)	0.462	0.535 (0.128–2.239)	0.392
Stage IIB	117	2.603 (1.186–5.711)	**0.017** ^a^	0.666 (0.051–8.737)	0.757
Stage III + IV	7	1.734 (0.442–6.799)	0.430	0.861 (0.139–5.347)	0.873
Grade	174				
G1+G2	124	Reference			
G3+G4	50	1.523 (0.986–2.352)	0.058		
Alcohol drinking history	164				
No	64	Reference			
Yes	100	1.125 (0.724–1.749)	0.601		
Site	176				
Body + tail + others	39	Reference			
Head	137	2.332 (1.291–4.213)	**0.005** ^a^	1.955 (1.037–3.686)	**0.038** ^a^
Tumor diameter	163	1.006 (0.899–1.126)	0.916		
Chemotherapy	176				
No	60	Reference			
Yes	116	0.582 (0.382–0.888)	**0.012** ^a^	0.395 (0.247–0.632)	**<0.001** ^a^

a p-value is less than 0.05.

**TABLE 2:** Independent prognostic factors for DFS in TCGA dataset.

Characteristics	Total (N)	Univariate analysis		Multivariate analysis	
		Hazard ratio (95% CI)	*p* value	Hazard ratio (95% CI)	*p* value
Risk score	69	35.806 (6.384–200.838)	**<0.001** ^a^	28.492 (3.902–208.069)	**<0.001** ^a^
Age (years)	69				
<65	31	Reference			
≥65	38	1.025 (0.447–2.349)	0.954		
Sex	69				
Male	37	Reference			
Female	32	2.372 (1.001–5.623)	0.050	2.055 (0.834–5.062)	0.118
N	66				
N0	23	Reference			
N+	43	3.881 (1.301–11.576)	**0.015** ^a^	1.160 (0.360–3.744)	0.803
T	68				
T1+T2	17	Reference			
T3+T4	51	2.379 (0.789–7.174)	0.124		
Stage	68				
Stage I	12	Reference			
Stage IIA	12	1.440 (0.197–10.498)	0.719		
Stage IIB	43	4.474 (1.010–19.821)	**0.049** ^a^		
Stage III+IV	1	0.000 (0.000-Inf)	0.998		
Grade	68				
G1+G2	51	Reference			
G3+G4	17	1.446 (0.593–3.521)	0.417		
Alcohol drinking history	64				
No	25	Reference			
Yes	39	1.116 (0.461–2.701)	0.808		
Site	69				
Body + tail + others	20	Reference			
Head	49	3.799 (1.259–11.463)	**0.018** ^a^	2.381 (0.799–7.095)	0.119
Tumor diameter	61	0.896 (0.643–1.249)	0.517		
Chemotherapy	69				
No	19	Reference			
Yes	50	2.111 (0.625–7.131)	0.229		

a p-value is less than 0.05.

### Validation of the IRGS in the External Cohort

**FIGURE 3:**
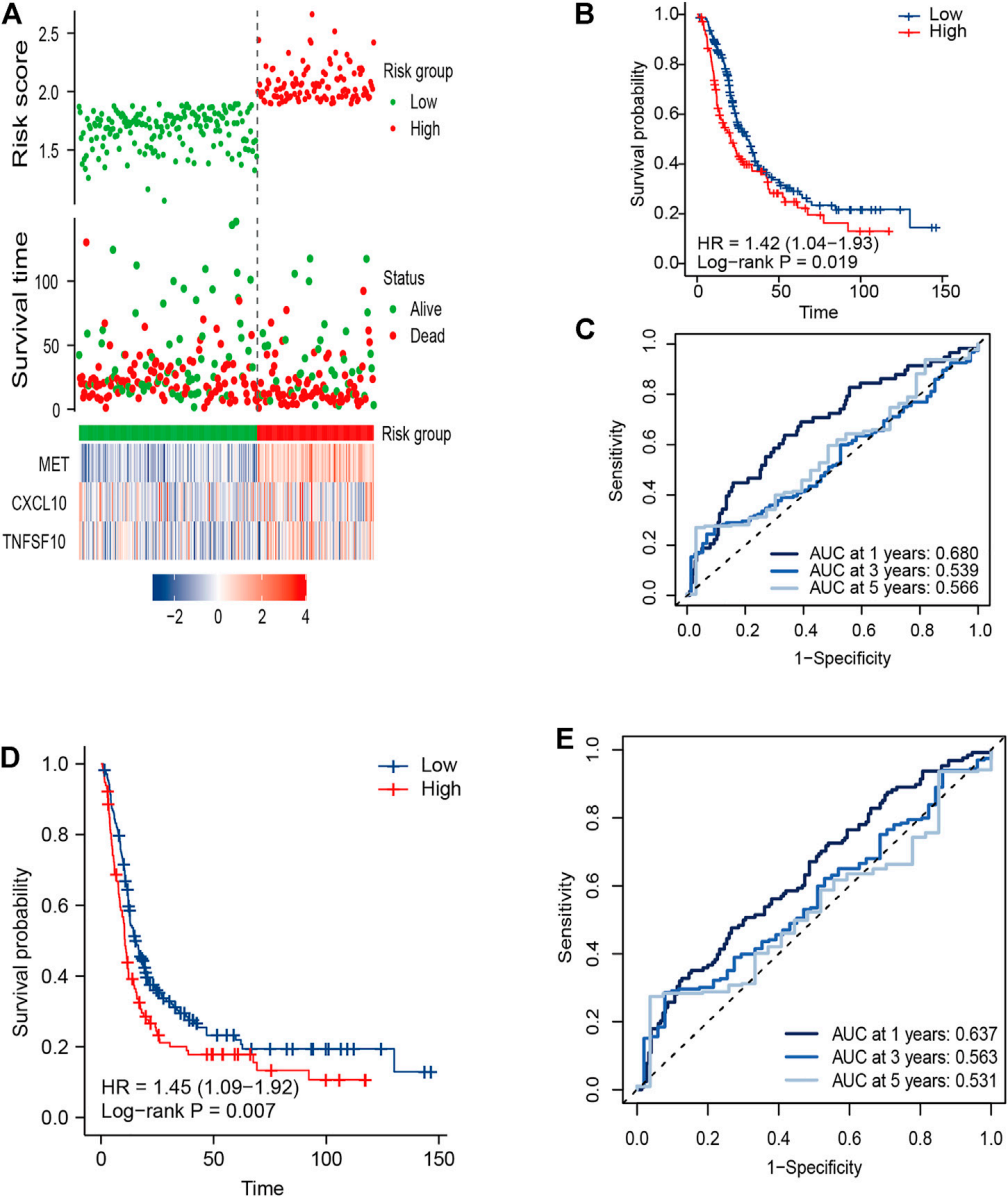
External validation of the three-gene signature in the E-MTAB cohort. **(A)** Distribution of the risk score, related survival status, and heatmap in the E-MTAB cohort. **(B,D)** KM survival curves for **(B)** OS and **(D)** DFS in the E-MTAB cohort. **(C,E)** Time ROC curves for forecasting 1-, 3-, and 5-year **(C)** OS and **(E)** DFS.

To validate the predictive capacity of the IRGS, the same formula was applied in the E-MTAB external validation cohort that included 288 PC patients with full survival information. Patients were divided into high-risk (*n* = 114) and low-risk groups (*n* = 174) by the same cutoff value derived from TCGA cohort, and the risk score distribution and live status are presented in Figure 3A. Expectedly, the Kaplan–Meier survival curves were significantly different in OS and DFS between the high- and low-risk groups, with worse outcomes for high-risk patients than low-risk patients (Figures 3B,D). The prognostic capacity of OS and DFS was subsequently evaluated by time-dependent ROC curves. For the validation cohort, the three-gene signature exhibited an acceptable performance for predicting 1-, 3- and 5-year OS, with an AUC value of 0.680, 0.539, and 0.566, respectively (Figure 3C). Comparable prediction performance in terms of 1-, 3- and 5-year DFS is shown in Figure 3E.

### Association Between the IRGS and Clinical Factors

**FIGURE 4:**
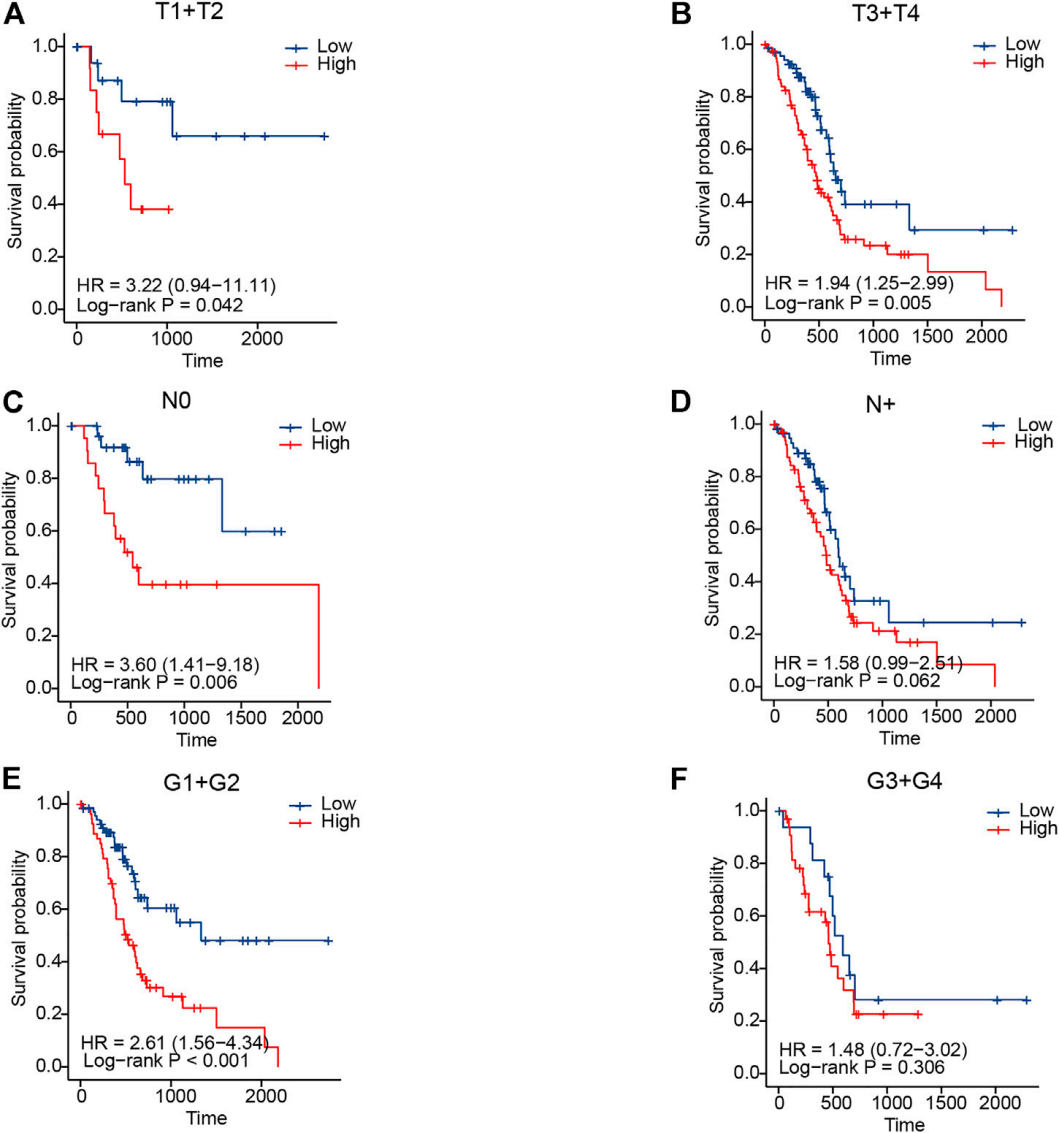
Association between the signature and clinical parameters in TCGA cohort. **(A,B)** KM curves for T-staging subgroups in high- and low-risk patients, respectively. **(C,D)** KM curves for lymph node metastasis status subgroups in high- and low-risk patients, respectively. **(E,F)** KM curves for tumor grade subgroups in high- and low-risk patients, respectively.

In clinical practice, the American Joint Committee on Cancer (AJCC) TNM staging system is used to assess the disease prognosis of patients, which, despite identification as a classification, still provides variable clinical outcomes because of the high molecular heterogeneity in PC. Accordingly, we tried to evaluate whether this novel IRGS could identify a subgroup of patients with an identical clinical parameter. For example, for patients with the same T-staging status, it was shown that the high-risk patients exhibited a worse OS than those in the low-risk group (*p* < 0.05 for both; Figures 4A,B).

Similar results were also observed in high-risk patients when stratified by lymph node metastasis status and tumor differentiation grade (Figures 4C–F). In the validation cohort, we also observed a similar result in patients when classified by different clinicopathological features (Supplementary Figure 1). The aforementioned results revealed that the IRGS-based stratification could further distinguish the prognosis, which might be a meaningful complement to the traditional TNM staging system.

### Relationship Between the IRGS and Drug Response in PC

There is no standard tool that can identify patients who are sensitive to chemotherapy. Accordingly, we tried to evaluate whether this novel IRGS could identify the subgroup of patients that are sensitive to chemotherapy. Considering the wide application of gemcitabine as chemotherapy for PC, we used the “pRRophetic” algorithm to assess the IC50 of samples based on the transcriptome data.

**FIGURE 5:**
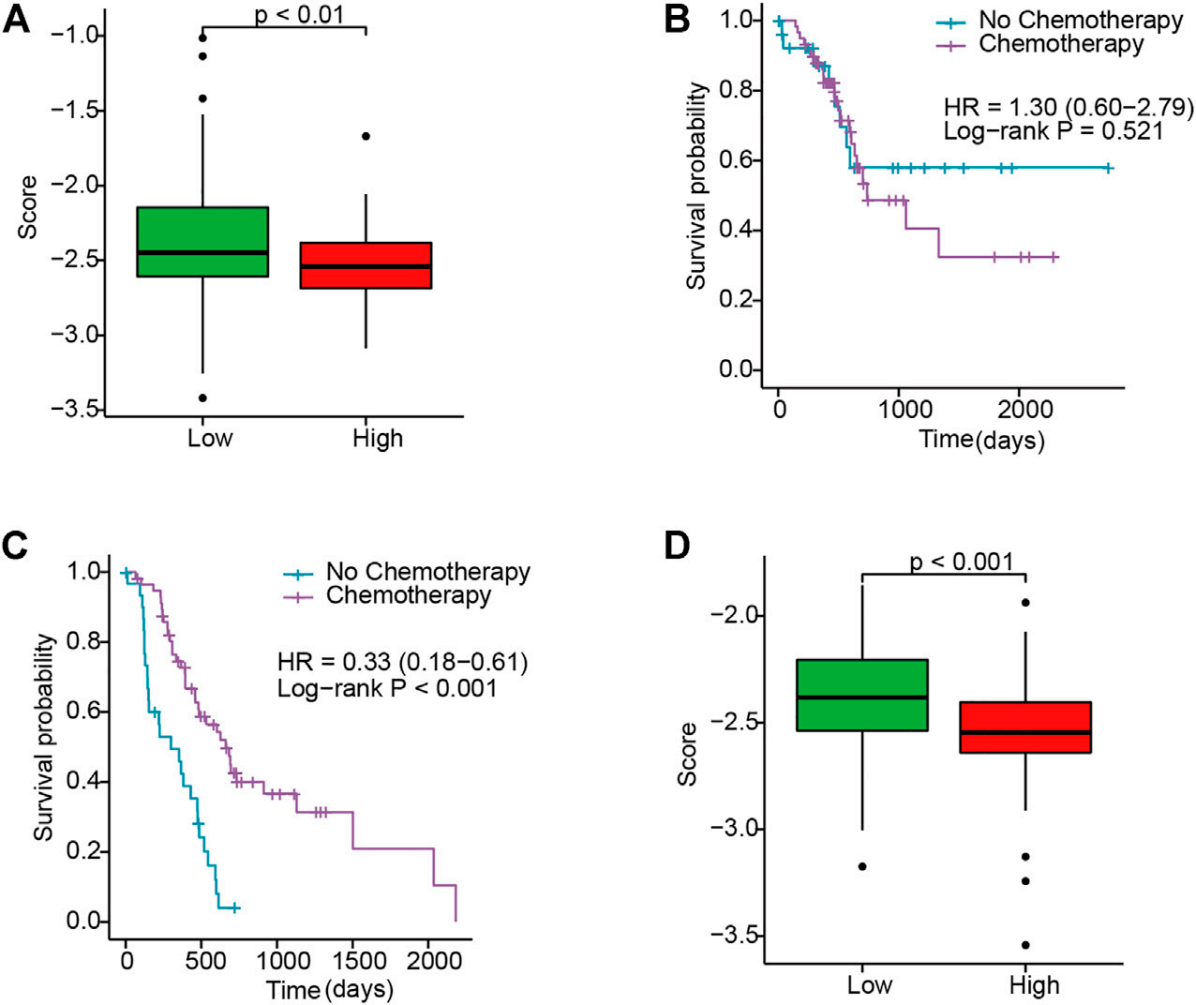
Evaluating the response to gemcitabine between high- and low-risk patients. **(A)** Box plot of the IC50 in TCGA. **(B,C)** KM curves for chemotherapy use subgroups in low- and high-risk patients in TCGA, respectively. **(D)** Box plot of the IC50 in E-MTAB.

After analyzing the IC50 for samples in TCGA and E-MTAB cohorts, the results revealed that there were conspicuous response sensitivities for high-risk patients as compared to low-risk patients (*p* < 0.01; Figures 5A,D). Moreover, compared with the low-risk group with no significant differences in OS, the Kaplan–Meier curves revealed higher survival in the high-risk group when stratified by chemotherapy use in TCGA cohort (*p* < 0.001; HR, 0.33; 95% CI, 0.18–0.61; Figures 5B,C). This indicated that the IRGS could act as a prognostic predictor and also potentially predict the benefit of chemotherapy for PC patients.

### Correlation Between the IRGS and Immune Infiltration

**FIGURE 6:**
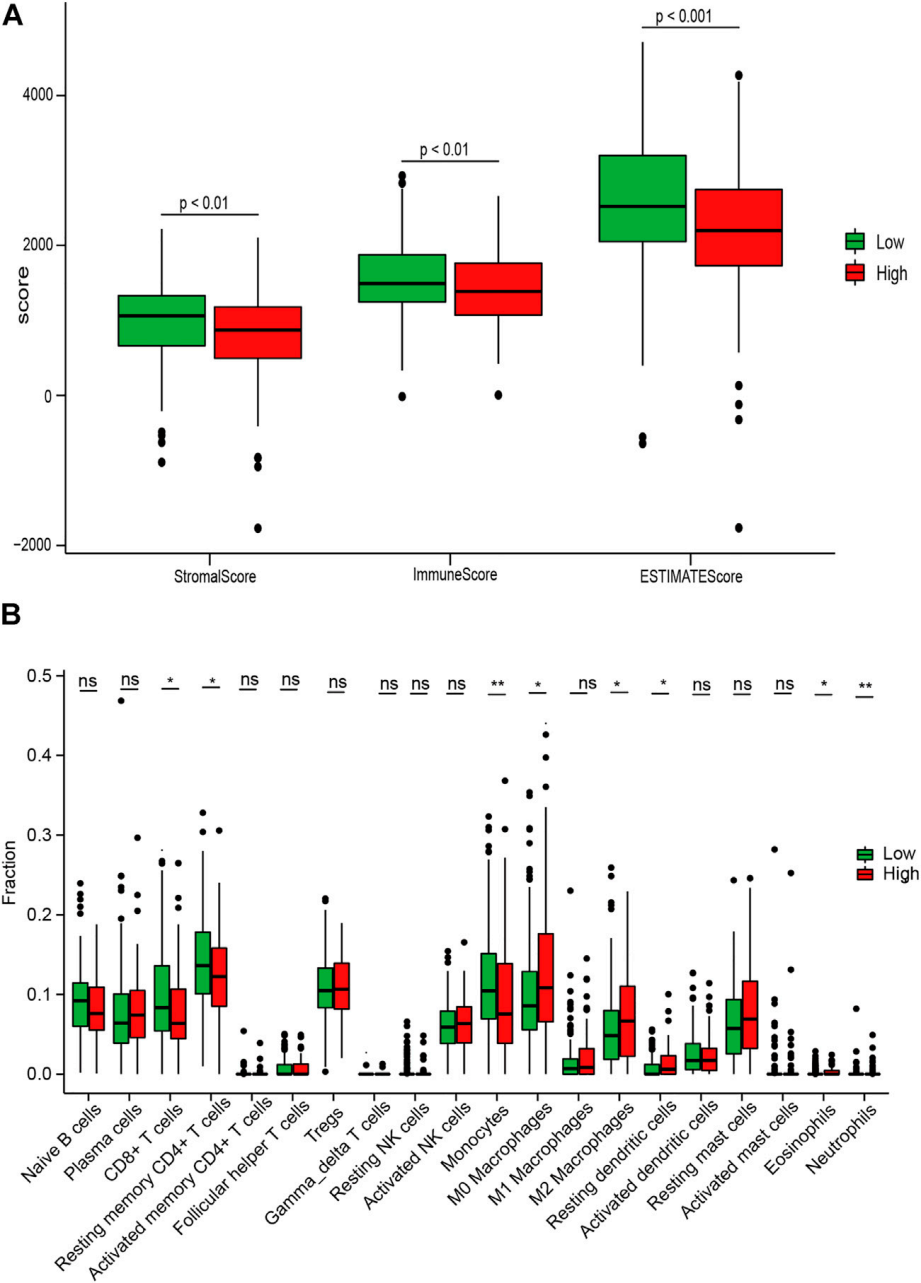
Associations between the IRGS and the tumor microenvironment. **(A)** Relationship between the risk score and the ESTIMATE score, stromal score, and immune score. **(B)** Distribution level of 22 tumor-infiltrating immune cells in the high- and low-risk groups. ns, no significance, *p < 0.05, **p < 0.01, ***p < 0.001.

Due to the crucial role of the TME components in immunotherapy response and survival prognosis, we analyzed the TME between the high- and low-risk sets based on the CIBERSORT and ESTIMATE algorithms. To confirm the correlation between the IRGS and the TME, we found that there was a significantly higher immune score, stromal score, and ESTIMATE score by box plot (*p* < 0.01; Figure 6A) for the low-risk set. Consistently, the results also revealed that low-risk patients were characterized by greater CD8^+^ T-cell infiltration, while there was greater M2 macrophage infiltration in high-risk patients (*p* < 0.05; Figure 6B).

Afterward, to clarify the association between the IRGS and the five molecular subtypes in PC (Puleo et al., 2018), the chi-square test was performed. The result suggested that patients in the high-risk group were more likely to possess a stroma-activated subtype as compared to those in the low-risk group (Table 3), which had been proved to have higher numbers of fibroblasts and lower immune cell infiltration in the TME. These results may assist in explaining why shorter survival times occurred in the high-risk group.

**TABLE 3:** Association between the IRGS and five molecular subtypes.

Molecular subtypes	Group		*p* value
	High risk	Low risk	
Desmoplastic	16	64	<0.0001
ImmuneClassical	4	31	0.0006215
PureBasal-like	28	6	<0.0001
PureClassical	33	57	0.6024
StromaActivated	41	29	0.0003487
Total	122	187	<0.0001

### Functional Enrichment Analysis of DEGs Between Different Risk Groups

**FIGURE 7:**
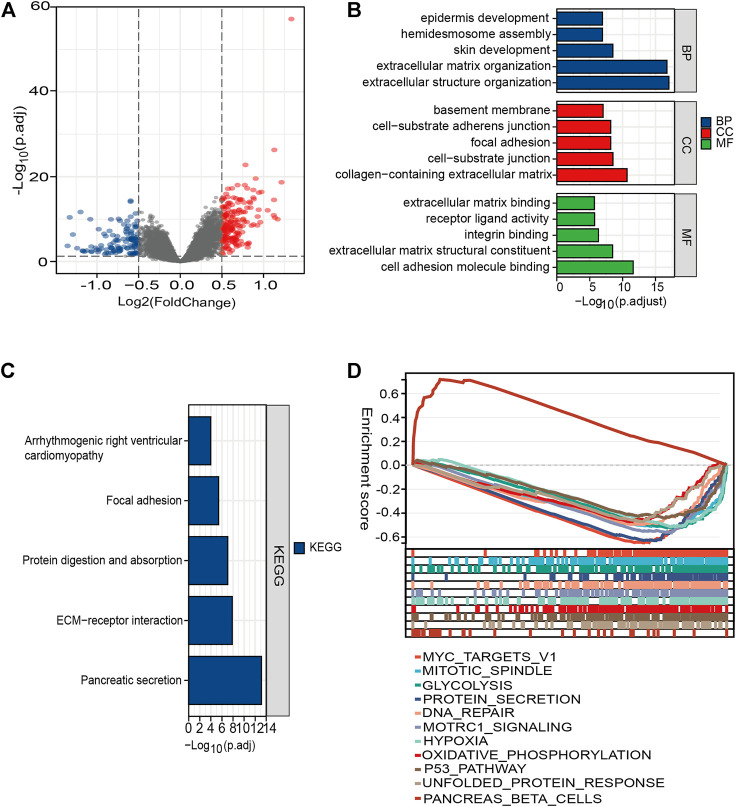
Functional enrichment analysis of the DEGs between different risk groups. **(A)** Volcano plot of genes. Blue dots represent downregulated expressed genes, while red dots represent upregulated expressed genes. **(B)** Top five classes of GO enrichment terms for genes in biological process (BP), cellular component (CC), and molecular function (MF). **(C)** Meaningful KEGG enrichment terms among genes. **(D)** Enriched gene sets in high- and low-risk groups by GSEA.

We found 307 DEGs between the high- and low-risk patients, of which 194 were upregulated and 113 were downregulated (Figure 7A and Supplementary Table 3). The functions of these genes were explored using GO and KEGG pathway enrichment analyses. Significantly enriched pathways were myofibroblast-related signaling pathways, such as extracellular structure, extracellular matrix structural constituent, and extracellular matrix organization (Figure 7B). This finding was consistent with the former observation that high-risk patients tended to be a stroma-activated molecular subtype.

The KEGG results demonstrated that the DEGs were involved in pathways (extracellular matrix (ECM)–receptor interaction, protein digestion) related to cellular dissociation *in situ* (Figure 7C; Supplementary Table 4). For H: hallmark gene collection defined by MSigDB, ten pathways, such as “HALLMARK_MYC_TARGETS_V1” and “HALLMARK_ HYPOXIA,” were significantly enriched in the high-risk group. However, only one pathway was enriched in the low-risk group (Figure 7D). Our results suggested that molecular alteration in the high-risk PC patients might be closely correlated with dense stroma reaction and a harsh TME, ultimately underlying a poorer clinical outcome.

## Discussion

Among common malignancies, pancreatic cancer (PC) bears the worst 5-year survival with remarkably dismal outcomes. Targeted therapies and immunotherapy are not yet available to improve clinical outcomes in unselected subjects, thus allowing chemotherapy to be the first-line therapeutic option for systemic therapy associated with superior long-term survival (Nicolle et al., 2021). Nevertheless, evidence from current practice suggests that some resectable patients have experienced disease progression during neoadjuvant chemotherapy and thus have lost the opportunity for consequential surgery, and there are no validated biomarkers that can be used to predict the clinical response to chemotherapy (Springfeld et al., 2019).

Although the AJCC staging system is now widely used for prognostic estimation of cancer, it often provides useful but inadequate prognostic information due to the high heterogeneity among PC. Additionally, despite efforts to identify many diagnostic and prognostic molecular markers, they have rarely been shown to have clinical application (Harsha et al., 2009; Yachida et al., 2010). Therefore, additional prognostic biomarkers are necessary to enhance the effectiveness of prognostic prediction. The current study attempted to develop a new signature that can be used to reliably identify patients that will benefit from chemotherapy and assess the prognosis to optimize personalized treatment.

The dense desmoplastic stroma in PC is believed to serve as a barrier for immune cell infiltration, and thus, it subsequently promotes cancer progression. The fibrotic process in the pancreas itself consists of several factors, such as tissue damage, chronic pancreatitis, and abnormal cell death. Such tissue damage activates the inflammation response at sites of damage, which is accompanied by cell multiplication and tissue remodeling (Barcellos-Hoff et al., 2013). Inflammatory response activates pancreatic stellate cells, which are responsible for the excess accumulation of the ECM, and ultimately promotes the metastasis and progression of PC (Pang et al., 2017).

Inflammatory response-related serum biomarkers such as neutrophils, platelets, lymphocytes, and monocytes have also shown satisfactory performance in predicting the prognosis of PC (Lu et al., 2020). Nevertheless, the inflammation response-related gene signature as a prognostic indicator for PC had rarely been studied. To this end, the focus was on inflammatory response-related genes because it was reported that they were related to cancer prognosis and TME characteristics, which have also been associated with chemotherapy effect in PC (Springfeld et al., 2019; Liu et al., 2020). Subsequently, we obtained a total of 27 candidate DEGs from 200 inflammatory response-related genes between PC and normal samples through differential expression analysis for further exploration.

To overcome the instability caused by tumor heterogeneity, a combination of biomarkers may be more stable in estimating PC prognosis than a single marker. Therefore, a novel three-gene signature (IRGS) produced by the LASSO-Cox method for survival prediction of PC was established. To the best of our knowledge, the three-gene prognostic signature described herein has not been previously reported. Previous studies reported that a nine-gene signature, hypoxia gene signature, and ferroptosis-related gene signature were used to estimate 1-year OS for PC with an AUC at 0.544, 0.602, and 0.7 (Wu et al., 2019; Ding et al., 2021; Jiang et al., 2021), respectively, which resembled the results of our study. In addition to the satisfactory survival prediction ability and construction using a small number of genes, this inflammatory response-associated gene signature we developed exhibits additional advantages over the above signature. For instance, it can distinguish immune microenvironment characteristics and chemotherapeutic agent sensitivity for high-risk and low-risk groups, and the risk score has been shown to correlate with gemcitabine sensitivity. The IRGS can also be used to stratify patients into subgroups with significantly different OS and DFS even with the same T- and N-staging or other clinicopathological features. The prognostic value of the three-gene signature was verified in the external E-MTAB cohort, and this signature was an independent risk factor for OS and DFS of PC.

The LASSO-Cox regression algorithm for the 27 DEGs identified a total of three core prognostic-related genes (CXCL10, TNFSF10, and MET) by integrative analysis of the openly accessible dataset. Of note, all the DEGs we identified were observed to be dramatically upregulated in PC samples, which was consistent with previous findings (Lin et al., 2021). CXCL10 is a chemokine that was initially identified as belonging to a cytokine subfamily that is primarily involved in the response of immune cells. It has been elucidated that chemokine expression in tumors plays a role in aggressiveness, immunosuppression of the TME, and drug resistance (Goto and Liu, 2020).

For example, recent studies have shown that the overexpression of CXCL10 can migrate tumor cells through the AKT and MEK signaling pathways to neurons, resulting in PC-associated neural invasion, and that chemokine blockers diminish this effect in an animal model (Hirth et al., 2020). Previous studies have also reported that CXCL10 exhibits anti-neoplasm activity by inhibiting angiogenesis and increasing the fraction of TIICs. Nonetheless, CXCL10 can also recruit tumor-associated macrophages in colon cancer, which enhanced the progression of cancer and led to a poor outcome (Chen et al., 2020). Thus, CXCL10 may exert dual actions on various cancers.

Because of the multifaceted effect of CXCL10 on the biological behavior of various cancers, it is challenging to utilize CXCL10 as a therapy target. TNFSF10, the superfamily member 10 of tumor necrosis factor (TNF), acts in an antitumor capacity by inducing cancer cell apoptosis while interacting with the corresponding receptor (Qu et al., 2019). However, De Looff et al. noted that complex TME–tumor biological interactions would impede the tumor reduction effect of therapeutic TNFSF10 receptor agonists (de Looff et al., 2019). It is, therefore, necessary to gain insight into the effect of different TME components on TNFSF10 activation before formal clinical application.

The mesenchymal–epithelial transitional tyrosine kinase receptor (Met) is encoded by the MET oncogene located on human chromosome 7 and is a member of the receptor tyrosine kinase (RTK) family. The binding of MET and its ligand causes the appearance of the characteristic hallmarks of cancer, including cell proliferation, inhibition of apoptosis, and invasion and metastasis, by activating downstream signaling pathways (Van Der Steen et al., 2016). However, targeted therapies for MET yield variable results with different tumor types, and clinical trials with different cancer types have shown variable anti-cancer effects (Spigel et al., 2017; Wakelee et al., 2017; Bradley et al., 2018). These results suggest that the paradoxical role of core genes in cancer progression warrants further exploration of the underlying mechanisms. Unlike most previous reports that focused on one inflammatory response-related gene, the present study explored the uncertain roles of these genes in PC utilizing integrative analysis.

To further understand the relationship between the risk score and the TME, we investigated the contribution of risk score to immune cell infiltration. We found that there were a greater amount of M2 macrophage infiltration and less CD8^+^ T-cell infiltration in the high-risk group, as well as a lower immune score. We also found that, for patients in the high-risk group, a stroma-activated molecular type was found with greater frequency, and it served as a barrier against antitumor immune cell infiltration and was associated with a worse prognosis (Erkan et al., 2012). These results suggest that immune surveillance is dysregulated in the high-risk group and that this may be an important reason for its poor prognosis.

Functional enrichment analysis revealed that DEGs between different groups were mainly enriched in the extracellular matrix and might perform biological functions through the interaction of the TME and tumor cells. According to the results of GSEA, the p53 pathway and several energy metabolism-related signaling pathways such as glycolysis, hypoxia, and oxidative phosphorylation were significantly enriched in the high-risk group. Sustained activation of these pathways in tissue was associated with PC, and thus, that might be a potential therapeutic target.

Nonetheless, there are a few shortcomings associated with this study. First, this research was retrospectively analyzed, and thus, additional prospective studies are required to examine the prognostic value of the gene signature in PC before clinical application. Second, additional experiments are required to elucidate the underlying mechanisms of the hub genes in TME regulation and progression of PC.

## Conclusion

Through systematical bioinformatics analyses, we constructed a novel inflammatory response gene signature with powerful predictive performance of survival and therapy response in PC, which may serve as an important supplement to the current AJCC staging and improve individualized treatment in the era of precision medicine. The three-gene signature was closely associated with regulation of immune cell infiltration, and the desmoplastic stroma of PC and its constituents could be candidates for therapeutic targets.

## Data Availability

Publicly available datasets were analyzed in this study. These data can be found here: TCGA database (https://portal.gdc.cancer.gov), ArrayExpress database (https://www.ebi.ac.uk/arrayexpress/experiments/E-MTAB6134/), and GTEx database (https://gtexportal.org/).

## Supplementary Material

Supplementary Figure 1 Association between the signature and clinical parameters in the E-MTAB cohort. **(A,B)** KM curves for T-staging subgroups in high- and low-risk patients, respectively. **(C,D)** KM curves for lymph node metastasis status subgroups in high- and low-risk patients, respectively. **(E,F)** KM curves for resection margin status subgroups in high- and low-risk patients, respectively.
