# Supplementary figures and images for: An Inflammatory Response-Related Gene Signature Reveals Distinct Survival Outcome and Tumor Microenvironment Characterization in Pancreatic Cancer
Source: Front Mol Biosci. 2022 Jun 8;9:876607. doi: 10.3389/fmolb.2022.876607 (PMC9216734; doi:10.3389/fmolb.2022.876607)

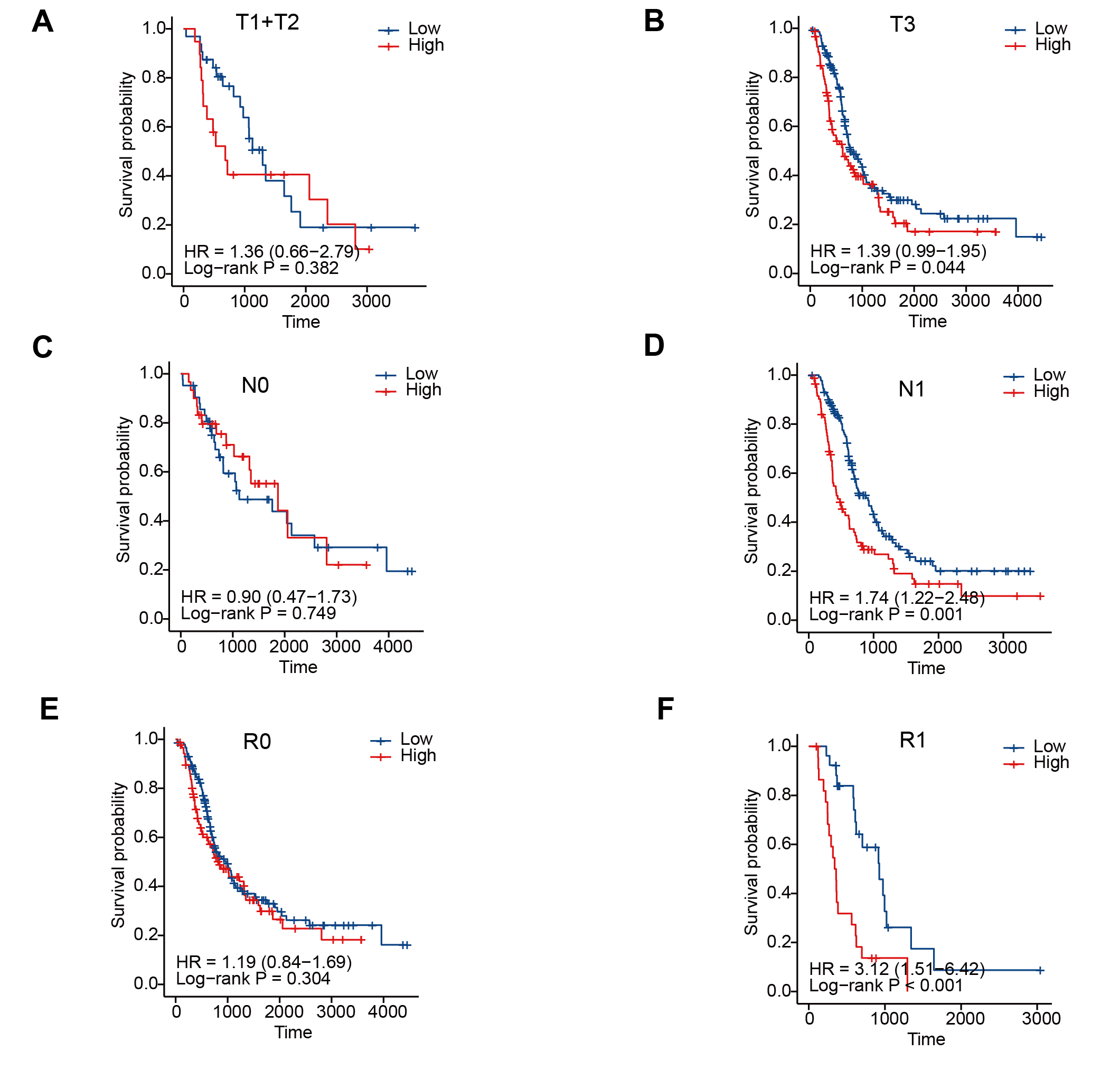

Supplement: Supplementary file 2 [file Image1.TIF]
