# Supplementary material for: An Inflammatory Response-Related Gene Signature Reveals Distinct Survival Outcome and Tumor Microenvironment Characterization in Pancreatic Cancer
Source: Front Mol Biosci. 2022 Jun 8;9:876607. doi: 10.3389/fmolb.2022.876607 (PMC9216734; doi:10.3389/fmolb.2022.876607)
Supplement: Supplementary file 3 [file Table3.DOCX]

**SUPPLEMENTARY TABLE 1 |** Independent prognostic factors for OS in the E-MTAB Dataset.

| Characteristics | Total(N) | Univariate analysis | |  | Multivariate analysis | |
| --- | --- | --- | --- | --- | --- | --- |
|  |  | Hazard ratio (95% CI) | P value |  | Hazard ratio (95% CI) | P value |
| Grade | 288 |  |  |  |  |  |
| G1 | 110 | Reference |  |  |  |  |
| G2 | 130 | 1.153 (0.834-1.595) | 0.389 |  | 1.150 (0.826-1.600) | 0.408 |
| G3 | 48 | 1.730 (1.139-2.626) | **0.010** |  | 1.674 (1.061-2.641) | **0.027** |
| T | 288 |  |  |  |  |  |
| T1 | 12 | Reference |  |  |  |  |
| T2 | 39 | 1.852 (0.762-4.503) | 0.174 |  |  |  |
| T3 | 237 | 1.918 (0.847-4.342) | 0.118 |  |  |  |
| N | 288 |  |  |  |  |  |
| N0 | 72 | Reference |  |  |  |  |
| N1 | 216 | 1.873 (1.296-2.705) | **<0.001** |  | 1.723 (1.175-2.526) | **0.005** |
| Resection margin | 283 |  |  |  |  |  |
| R0 | 234 | Reference |  |  |  |  |
| R1 | 49 | 1.872 (1.292-2.714) | **<0.001** |  | 1.638 (1.118-2.400) | **0.011** |
| Risk score | 288 | 1.892 (1.016-3.522) | **0.044** |  | 1.655 (0.843-3.248) | **0.043** |
