# Supplementary material for: An Inflammatory Response-Related Gene Signature Reveals Distinct Survival Outcome and Tumor Microenvironment Characterization in Pancreatic Cancer
Source: Front Mol Biosci. 2022 Jun 8;9:876607. doi: 10.3389/fmolb.2022.876607 (PMC9216734; doi:10.3389/fmolb.2022.876607)
Supplement: Supplementary file 5 [file Table4.DOCX]

**SUPPLEMENTARY TABLE 2 |** Independent prognostic factors for DFS in the E-MTAB Dataset.

| Characteristics | Total(N) | Univariate analysis | |  | Multivariate analysis | |
| --- | --- | --- | --- | --- | --- | --- |
|  |  | Hazard ratio (95% CI) | P value |  | Hazard ratio (95% CI) | P value |
| Grade | 288 |  |  |  |  |  |
| G1 | 110 | Reference |  |  |  |  |
| G2 | 130 | 1.269 (0.942-1.708) | 0.117 |  | 1.227 (0.905-1.664) | 0.188 |
| G3 | 48 | 1.640 (1.112-2.420) | **0.013** |  | 1.459 (0.949-2.245) | 0.085 |
| T | 288 |  |  |  |  |  |
| T1 | 12 | Reference |  |  |  |  |
| T2 | 39 | 2.249 (0.930-5.435) | 0.072 |  | 1.778 (0.677-4.667) | 0.243 |
| T3 | 237 | 2.732 (1.209-6.173) | **0.016** |  | 2.002 (0.809-4.957) | 0.133 |
| N | 288 |  |  |  |  |  |
| N0 | 72 | Reference |  |  |  |  |
| N1 | 216 | 1.950 (1.393-2.729) | **<0.001** |  | 1.734 (1.215-2.475) | **0.002** |
| Resection margin | 284 |  |  |  |  |  |
| R0 | 235 | Reference |  |  |  |  |
| R1 | 49 | 2.173 (1.553-3.040) | **<0.001** |  | 1.802 (1.276-2.544) | **<0.001** |
| Risk score | 288 | 1.962 (1.129-3.410) | **0.017** |  | 1.874 (1.033-3.398) | **0.039** |
